# ParticipACTION: Baseline assessment of the 'new ParticipACTION': A quantitative survey of Canadian organizational awareness and capacity

**DOI:** 10.1186/1479-5868-6-86

**Published:** 2009-12-09

**Authors:** Ronald C Plotnikoff, Ivan Todosijczuk, Guy Faulkner, Michael A Pickering, Susan Cragg, Karen Chad, John C Spence, Mark Tremblay, Cora L Craig, Adrian Bauman, Larry Brawley, Lise Gauvin

**Affiliations:** 1 School of Public Health and the Faculty of Physical Education & Recreation, University of Alberta, Edmonton, Alberta, Canada; 2 School of Education, University of Newcastle, University Drive, Callaghan, NSW 2300, Australia; 3 Faculty of Physical Education and Recreation, University of Alberta, E488 Van Vliet Centre, Edmonton, AB, T6G 2H9, Canada; 4 Faculty of Physical Education and Health, University of Toronto, 55 Harbord Street, Toronto, ON, M5S 2W6, Canada; 5 Canadian Fitness and Lifestyle Research Institute, 201-185 Somerset Street West, Ottawa, ON, K2P 0J2, Canada; 6 College of Kinesiology, University of Saskatchewan, PAC 300 87 Campus Drive, Saskatoon, SK, S7N 5B2, Canada; 7 Healthy Active Living and Obesity Research Group, Children's Hospital of Eastern Ontario Research Institute, 401 Smyth Road, Ottawa, ON, K1H 8L1, Canada; 8 School of Public Health, University of Sydney, K25 - Medical Foundation Building, Sydney, NSW 2006, Australia; 9 Faculty of Medicine, Department of Social & Preventive Medicine, GRIS (Groupe de recherche interdisciplinaire en santé), Centre de recherche Léa-Roback sur les inégalités sociales de santé de Montréal, Université de Montréal, PO Box 6128, Downtown Station, Montreal, PQ., H3C 3J7, Canada

## Abstract

**Background:**

ParticipACTION is a Canadian physical activity (PA) communications and social marketing organization that was relaunched in 2007 after a six-year hiatus. This study assesses the baseline awareness and capacity of Canadian organizations that promote physical activity, to adopt, implement and promote ParticipACTION's physical activity campaign. The three objectives were: (1) to determine organizational awareness of both the 'original' and 'new' ParticipACTION; (2) to report baseline levels of three organizational capacity domains (i.e., to adopt, implement and externally promote physical activity initiatives); and, (3) to explore potential differences in those domains based on organizational size, sector and primary mandate.

**Methods:**

Organizations at local, provincial/territorial, and national levels were sent an invitation via email prior to the official launch of ParticipACTION to complete an on-line survey. The survey assessed their organization's capacity to adopt, implement and externally promote a new physical activity campaign within their organizational mandates. Descriptive statistics were employed to address the first two study objectives. A series of one-way analysis of variance were conducted to examine the third objective.

**Results:**

The response rate was 29.7% (268/902). The majority of responding organizations had over 40 employees and had operated for over 10 years. Education was the most common primary mandate, followed by sport and recreation. Organizations were evenly distributed between government and not-for-profits. Approximately 96% of respondents had heard of the 'original' ParticipACTION while 54.6% had heard of the 'new' ParticipACTION (Objective 1). Findings indicate good organizational capacity in Canada to promote physical activity (Objective 2) based on reported means of approximately 4.0 (on 5-point scales) for capacity to adopt, implement, and externally promote new physical activity campaigns. *Capacity to adopt *new physical activity campaigns differed by organizational sector and mandate, and *capacity to implement *differed by organizational mandate (Objective 3).

**Conclusion:**

At baseline, and without specific details of the campaign, respondents believe they have good capacity to work with ParticipACTION. ParticipACTION may do well to capitalize on the existing strong organizational capacity components of leadership, infrastructure and 'will' of national organizations to facilitate the success of its future campaigns.

## Background

ParticipACTION was originally launched in 1971 with financial support from the federal government. In essence, ParticipACTION was a social marketing organization dedicated to promoting physical activity and fitness in Canada. ParticipACTION produced many successful national physical activity campaigns and its name and brand is recognized by most adult Canadians [1]. Recently, other countries have had similar successful national initiatives: VERB™ in the United States of America [2], the 'Push Play' campaign in New Zealand [3], 'Get Moving' in Australia [4] and the Agita São Paulo program in Brazil [5].

Thirty years after its inception, ParticipACTION ceased operations in 2001 due to lack of funding (details of ParticipACTION's history and the mandate of the 'new' ParticipACTION can be found in Tremblay and Craig [6]). After 6 years of hibernation, the 'new' ParticipACTION was resurrected in 2007 as a social marketing organization "to inspire and support Canadians to move more" [7]. It plans to achieve this goal of motivating Canadians through campaigns, programs and initiatives developed with its partner organizations and sponsors. ParticipACTION's new role in the physical activity landscape is to act as a catalyst (stimulus) for communication and action with partners and stakeholders through a national communication voice and through the marshalling of resources to support the cause of physical activity promotion in Canada [7].

Although the original ParticipACTION's sustainability has been credited to its flexibility of operation and multi-sectoral capacity [8], evaluations of the original campaigns focused on individual awareness, recall, and understanding. Less studied has been the impact such campaigns have had on the broader organizational climate to mobilize and advocate for physical activity - an important 'upstream' outcome [9] for facilitating a more active population. The process of building capacity is itself a valuable dimension that fundamentally adds to the actual outcomes of health promotion [10]. Understanding and identifying current capacity of organizations to adopt and integrate new, externally generated campaigns into their mandates is essential. Importantly, ParticipACTION will not be involved in direct programming [7]. This raises a key question: do Canadian organizations involved in physical activity and/or health promotion have the capacity (defined below) to work with ParticipACTION on its campaign and to adopt and implement its initiatives? The operational hiatus between 2001 and 2007 has provided a unique, time sensitive opportunity to collect baseline information about the present state of organizational capacity to work with ParticipACTION.

The purpose of this study was to determine the capacity of Canadian organizations at local, provincial and national levels involved in physical activity and health promotion to work with ParticipACTION to achieve the goal of making Canada a more active country. The three objectives of this study were: (1) to determine organizational awareness of the 'original' and 'new' ParticipACTION; (2) to report baseline levels of three organizational capacity domains (to adopt, to implement and to externally promote a physical activity initiative); and (3) to explore potential differences in those domains based on: a) size, b) sector (i.e., government, not-for-profit, private), and c) primary mandate (i.e., public health, health care, sport, recreation, and education) of the organizations. Objective 3 also allows us to examine how an organization's size, sector or mandate might influence its ability to become involved with a new campaign. It must be noted that at the time this study was conducted, the specific details of the campaign were unknown. This information will facilitate the assessment of future campaigns and initiatives.

### Theoretical Background for the Study

Organizational capacity, and specifically health promotion capacity, has received considerable study. The Singapore Declaration: *Forging the Will for Heart Health in the Next Millennium *describes capacity as the quality in an individual or an organization to achieve and maintain change [11]. Capacity also includes the ability to address new problems based on the skills of the organization [11].

Organizational capacity has been conceptualized as comprising three key components: *leadership*, *infrastructure*, and *will *[12]. This study focuses on identifying current levels of *leadership, infrastructure *and *will *of an organization's capacity to work with ParticipACTION.

*Leadership *encompasses such constructs as decision-making, organizational climate, "champions" [12], and developing partnerships, collaborations and linkages within the community [13]. *Infrastructure *refers to organizational knowledge, human and financial resources, and organizational processes [12]. *Will *is defined by prior actions, planning, and setting priorities [12] as well as the predisposition or the motivation to undertake activities, such as health or PA promotion, and the belief of the importance of those activities [14]. An organization's capacity based on its leadership, will, and infrastructure should influence the extent to which ParticipACTION's activities can be adopted and implemented within those organizations.

Using Diffusion of Innovations (DIT) [15], RE-AIM Framework [16,17], and Interorganizational Relations Theory (IOR) [18] as our theoretical guides, we focused our study on key constructs within these theories as they apply to organizational capacity. In brief, DIT is defined as "the process by which an innovation is communicated through certain channels over time among members of a social system" ([15], page 5). This occurs through 5 stages, three of which are addressed in this study: (1) persuasion (dissemination and acquisition of information about an innovation); (2) decision (adoption or rejection of an innovation); and, (3) implementation of the innovation [15]. Each plays a role in an organization's capacity to work with ParticipACTION.

The RE-AIM framework [16,17], which was heavily influenced by Rogers DIT [15], proposes that population-based interventions should be evaluated along five dimensions. Three of these were addressed in the study: (1) reach (i.e., the segment of the target population affected by an intervention); (2) adoption (i.e., identifies the organizational and staff capabilities to offer a specific intervention or program); and (3) implementation (i.e., how closely staff, organizations, and settings adhere to the content of the intervention and its delivery).

IOR is a contingency theory. It proposes that increased complexity of health, political, social, and economic factors encourage or require organizations to form networks to effectively respond to a specific challenge such as increasing physical activity at the population level [19]. Since ParticipACTION will not provide actual programming to the public, it must work with existing organizations which can and do provide programming.

These theoretical models and frameworks provide insight into how ParticipACTION's social marketing efforts may be disseminated to and adopted by organizations in Canada. The evaluation of future initiatives requires identifying baseline awareness of physical activity and health promotion organizations about ParticipACTION and their capacity to adopt, implement and promote ParticipACTION campaigns. To our knowledge this is the first study to examine organizational awareness and organizational capacity prior to the official launch of a new national initiative. Since two of ParticipACTION's strategic goals are to (1) develop a legacy of collaboration and partnership with organizations across Canada, and (2) to set the stage for long-term sustainability of this physical activity movement [7], it is important to have a baseline assessment of the interest in and the ability to adopt, implement and promote campaigns led by ParticipACTION. This study will provide information about such collaboration and sustainability within Canada, across mandates and sectors. This information is also essential for any and all subsequent program and organizational evaluations surrounding ParticipACTION.

## Methods

### Sampling Frame

The sample included national alliances, networks and organizations at the local, provincial/territorial and national levels. In order to establish a comprehensive list of Canadian organizations which promote physical activity and which serve all ages and needs, we identified organizations as being involved in some form of physical activity promotion, whether through direct promotion, health promotion, education, or sport and recreation. These organizations included members of the 'Coalition for Active Living' (119 member organizations [MO's]), 'Chronic Disease Prevention Alliance of Canada' (50 MO's), 'Coaching Association of Canada' (68 MO's), 'Sport Matters' (59 MO's), boards of education across all provinces and territories (281 boards), provincial athletic associations (69 MO's), Quebec based sports groups and organizations (77 MO's), 'Canadian Association of Health, Physical Education, Recreation and Dance' (13 MO's), 'Public Health Agency of Canada' (40 individual contacts), 'Active Living Alliance for Canadians with a Disability' (47 MO's), fitness clubs and organizations (99 businesses), and manufacturers/retailers (32 companies). In total, 966 organizations were sent invitations to participate in the survey.

### Data Collection

An on-line instrument was created in both official languages (French and English) using *Survey Monkey*. This allowed respondents to complete the questionnaire in their language of choice. The questionnaire was developed by members of the research team and evaluated by external reviewers who assessed the design of the instrument for ease of access, navigation, and completion. The instrument was then modified based on feedback.

Invitations requesting participation in the study were sent via email with one additional reminder email based on a modified Dillman technique [20]. The invitations were sent to individuals identified as a key contact having knowledge of their organization (directors, program coordinators) that specified "the survey should be completed by a representative from your organization who has a good knowledge of your organization to provide us with the most accurate feedback possible." Where possible (approximately 90%), invitations were sent to a named individual within the organization with the email also requesting "if this invitation has arrived with the wrong person, would you please forward to the most appropriate individual in your organization."

The invitation provided a description of the study and information for informed consent. Invitees could either select the hyperlink embedded in the email, or copy/paste the web-link into their browser to access the survey at *Survey Monkey*. Upon accessing the survey, invitees indicated consent to participate, and chose whether or not to continue with the survey or to exit. Respondents could change answers if desired, and were able to complete the survey over a number of sessions. A final question invited respondents to participate in a follow-up qualitative study [21].

The initial invitations were emailed September 24, 2007, three weeks prior to the official launch of the first campaign of the 'new' ParticipACTION on October 15. The reminder was sent approximately two weeks later. Access to the survey website was closed November 30, 2007.

### Measures

*Organizational demographics *assessed organizational size (<10, 10 - 39, 40+ employees), years involved in PA or health care promotion, scope of activity (i.e., local, provincial, national), organizational sector (i.e., government, not-for-profit, private), and primary mandate (i.e., public health/health care, sport and recreation/recreation, education).

#### Awareness of ParticipACTION

Four single-item questions assessed organizational knowledge about the original and the new ParticipACTION (with "Yes/No" response options): "Have you ever heard of ParticipACTION?"; "Have you heard anything about ParticipACTION in the last 12 months?"; "Have you heard anything about the new ParticipACTION?"; "Are you aware of any ParticipACTION resources?"

#### Organizational Capacity Scales

The three organizational capacity scales (Appendix 1), with response options ranging from (1) "not at all" to (5) "very" assessed organizational capacity to: (1) adopt a new physical activity initiative (7-items; α = .89); (2) implement a new physical activity initiative (11-items; α = .90); (3) externally promote a new physical activity initiative (9-items; α = .90). These scales were modified from validated scales developed for the 'Alberta Heart Health Project' (AHHP) [12,13] that specifically assessed organizational *leadership *[22], *infrastructure *[23] and *will *[14].

### Analysis

Descriptive statistics were employed to address the first two study objectives (i.e., to assess awareness of ParticipACTION, both original and new, and to report baseline levels of the three organizational capacity domains). Due to small sample sizes in certain categories, and for a more parsimonious approach to address the second objective, we collapsed some of the categories (see footnote Table 1). To address the third objective (to explore potential differences of the three capacity domains), univariate, one-way analyses of variance and t-tests were conducted to examine capacity score differences on each of the three capacity domains (i.e., to adopt, implement, and externally promote a new physical activity initiative) by each of the three demographic characteristics (i.e., size, sector, mandate). Significance was set at p < 0.05.

**Table 1:** Responding organization characteristics

Organizational Characteristic	Frequency (n)	(%)
How many years has your organization been involved in promoting PA/health?		
Less than 5 years	7	(3.9%)
5-10	19	(10.6%)
11-15	20	(11.2%)
16-20	12	(6.7%)
More than 20 years	121	(67.6%)
**Total**	179	100%
Do you work mainly at the national, provincial or territorial, or local level?		
national	64	(29.6%)
provincial/territorial	66	(30.6%)
local	75	(34.7%)
other	11	(5.1%)
**Total**	216	100%
	
How many people are there in your organization who work full time?		
Less than 10 employees	67	(38.1%)
10-39 employees	27	(15.3%)
40 or more employees	82	(46.6%)
**Total**	176	100%
^1 ^Do you work in the government, not-for-profit or private sector?		
Government	87	(47.5%)
Not-for-profit	93	(50.8%)
Private	3	(1.6%)
**Total**	183	100%
^2^What is the primary mandate of your organization?		
Public health/Health Care	19	(10.3%)
Sport and Recreation/Recreation	63	(34.1%)
Education	89	(48.0%)
Urban Planning, Transportation	0	(0.0%)
Other	14	(7.6%)
**Total**	185	100%

Note: Analysis for Objectives 2 & 3:

1. Organizational sector: post-secondary (n = 1) and education (n = 40) were combined with government since all educational facilities are affiliated with provincial education and school boards. The private sector (n = 3) was not included in the analysis.

2. Organizational mandate: public health (n = 12) and health care (n = 7) were combined; recreation (n = 8) and sport and recreation (n = 55) were combined; education (n = 89) was left unchanged. Urban planning (n = 0), transportation (n = 0) and other (which was undefined) (n = 14) were omitted from the analysis.

## Results

### Response Rate

**Figure 1:**
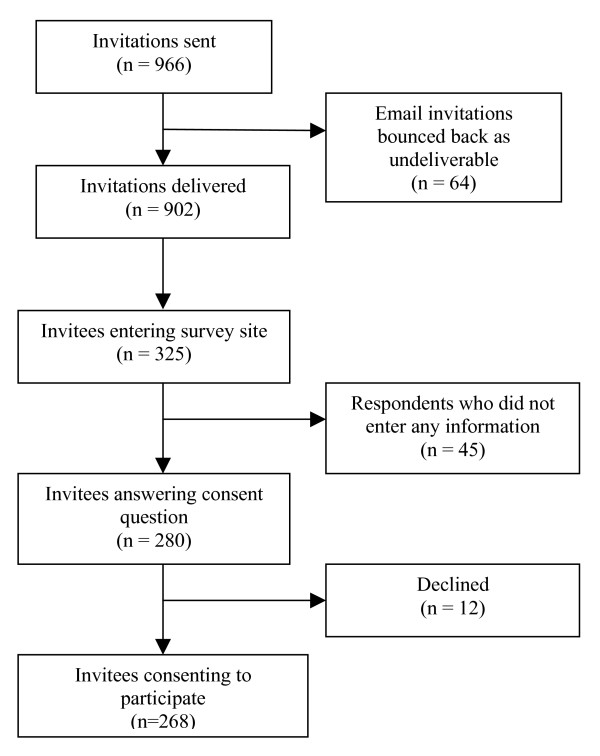
**Flow Chart of participation of organizations throughout the research project**.

Figure 1 illustrates the study flow and the response rate for the survey. A total of 966 email invitations were sent. Sixty-four (64) invitations were returned as being undeliverable. Of 902 delivered to intended respondents, we were not able to determine whether non-respondents had opened or read their invitations. A total of 325 invitees accessed the survey tool. Of those, 280 answered the "consent to participate" question, with 268 agreeing to participate and 12 declining. Therefore the response rate for those receiving an invitation and taking part in the survey was 29.7% (268/902).

As presented in Table 2, organizations from most provinces and territories responded in numbers which are relatively representative of the national population. The notable exception was Quebec with a very low response rate compared to its population. By mandate, the highest response rate came from Education with the lowest from Public Health/Health Care (Table 3).

**Table 2:** Survey response rate by province

Response Rate by Province		
**Province/Territory**	**% response of total sample**	% of Canadian population*
		
British Columbia	9.7	13.0
Alberta	15.4	10.4
Saskatchewan	6.3	3.1
Manitoba	9.1	3.6
Ontario	37.7	38.4
Quebec	4.6	23.8
New Brunswick	5.7	2.3
Nova Scotia	5.2	2.9
Prince Edward Island	1.7	0.4
Newfoundland/Labrador	1.7	1.6

*Based on Census figures for 2006 (Statistics Canada)

**Table 3:** Survey response rate by primary mandate

Response Rate by Mandate		
**Primary Mandate**	**% of invitees**	**% response of total sample**
		
Education	31.0	48.0
Sport/Recreation	24.0	34.1
Public Health/Health Care	41.0	10.3
Private (fitness clubs, retailers, manufacturers)	4.0	3.0
Other (undefined)	--	4.6
	100.0	100.0

### Sample Characteristics

The organizational demographic characteristics are displayed in Table 1. The majority of organizations (85%) have been operating for over 10 years with about 68% operating for over 20 years. There is a fairly even distribution (almost 30% each) at the local, provincial/territorial and national levels. The majority of respondents were from organizations with 40 or more employees (46.6%) while 38.1% reported working in organizations with fewer than 10 employees. Slightly over half (50.8%) of respondents indicated working in the not-for-profit sector; 47.5% indicated working within the government. Just under half (48%) listed education as their primary mandate with approximately one third (34.1%) listing sport and/or recreation as their primary mandate.

### Awareness of Participation (Objective 1)

Approximately 96% of respondents indicated they had heard of ParticipACTION; 54.6% had heard of the 'new' ParticipACTION. Of those that had heard of the 'new' ParticipACTION, 29.0% (15% of all respondents) indicated they were aware of ParticipACTION resources (i.e., posters, website, toolkits).

### Levels of three organizational capacity domains (Objective 2)

Table 4 shows capacity levels (to adopt, implement and externally promote a new PA initiative) by organizational size, sector and organizational mandate. Organizations reported means of approximately 4.0 (on 5-point scales) across the three capacity domains by each of the three demographic characteristics.

**Table 4:** Organization demographic group mean scores on three capacity domains.

	n range^1^	Capacity to Adopt PA Initiative Mean (SD)	Capacity to Implement PA Initiative Mean (SD)	Capacity to Externally Promote PA Initiative Mean (SD)
Organizational Size				
< 10 employees	56-67	3.96 (0.60)	3.90 (0.57)	3.81 (0.62)
10-39 employees	24-27	3.81 (0.82)	4.08 (0.64)	3.78 (0.69)
40 + employees	77-82	3.95 (0.74)	4.05 (0.68)	3.85 (0.89)
**Total**		**3.93 (0.70)**	**4.00 (0.64)**	**3.83 (0.77)**
		F (2, 154) = 0.42 p = .655	F (2, 173) = 1.35 p = .262	F (2, 165) = 0.12 p = .890
				
Organizational Sector				
Government (including all education)	78-87	4.06 (0.66)	4.01 (0.67)	3.88 (0.85)
Not-for-profit	87-93	3.83 (0.73)	3.99 (0.58)	3.70 (0.79)
**Total**		**3.94 (0.70)**	**4.00 (0.62)**	**3.79 (0.83)**
		t(158) = 2.06 ^2a^p = .041	t(178) = 0.26 p = .794	t(171) = 1.46 p = .147
				
Organizational Mandate				
Public health, health care	17-19	3.70 (0.89)	3.95 (0.76)	3.83 (0.89)
Sport & recreation, recreation	55-63	3.93 (0.63)	3.82 (0.59)	3.68 (0.80)
Education	80-89	4.08 (0.59)	4.08 (0.63)	3.90 (0.82)
**Total**		**3.98 (0.65)**	**3.99 (0.64)**	**3.81 (0.82)**
		F (2, 149) = 2.81 ^2b^p = .063	F (2, 168) = 3.19 ^2c^p = .044	F (2, 162) = 1.19 p = .306

Notes:

^1^Group sizes vary depending on response rate to each of the three scales.

^2^. Significant differences/trends exist

a - between governmental and not-for-profit organizations in capacity to adopt (p = .041; η^2 ^= .026)

b - between educational and public health/health care organizations in capacity to adopt (p = .063; η^2 ^= .036)

c - between education and sport & recreation/recreation in capacity to implement (p = .044; η^2 ^= .037)

### Organizational characteristic differences amongst the organizational capacity domains (Objective 3)

Although the findings did not reveal any specific patterns, we did find differences within organizational capacities (Table 4). Organizational capacity *to adopt a new physical activity initiative*, such as ParticipACTION, did not differ by organizational size [F (2, 154) = 0.42, p = .655]. There was a difference by organizational sector with Government showing a higher capacity to adopt (M = 4.06, SD = 0.66) than Not-for-Profit (M = 3.83, SD = 0.73), [t (158) = 2.06, p = .041, η^2 ^= .026]. When comparing organizational mandate, Education had a higher mean score (M = 4.08, SD = 0.59) than Public Health/Health Care (M = 3.70, SD = 0.89), [F(2, 149) = 2.81, p = .063, η^2 ^= .036], approaching significance with a p-value of 0.067. All effect sizes (η^2^) observed for significant differences were small, according to Cohen's guidelines [24].

Organizational capacity *to implement a new physical activity initiative *did not differ by organizational size [F (2, 173) = 1.35, p = .262], or by sector [t(178) = .26, p = .794]. It did show a significant difference for organizational mandate. Education had a higher mean score (M = 4.08, SD = 0.63) than did Sport and Recreation/Recreation (M = 3.82, SD = .059) [F(2, 168) = 3.188, p = .044, η^2 ^= .037)] [24].

Organizational capacity *to externally promote a new physical activity initiative *did not significantly differ by any of the three organization characteristics, organizational size [F (2, 165) = .12, p = .890], organizational sector [t(171) = 1.46, p = .147], or organizational mandate [F (2, 162) = 1.19 p = .306].

## Discussion

This study assessed the baseline awareness of Canadian organizations at the local, provincial/territorial and national levels regarding the 'original' and 'new' ParticipACTION and assessed their capacity to work with ParticipACTION to promote physical activity.

The first objective, determining organizational awareness of both the 'original' and 'new' ParticipACTION, varied amongst respondents. Most respondents (96.4%) had heard of the 'original' ParticipACTION. This suggests a strong and ongoing cultural memory for the organization brand. This result is not surprising since the majority of organizations (85%) have operated for over 10 years (68% over 20 years). Familiarity with 'new' ParticipACTION was lower at 54.6%. Although the official launch was in October 2007, a public announcement was made in February 2007 (6-months prior to ParticipACTION's baseline assessment and becoming operational). It could be expected that knowledge of the new program based on a public announcement would have diffused through these organizations via unofficial as well as official channels. Even fewer of the organizations, 15% of total respondents (or 29% of those familiar with the 'new' ParticipACTION) were aware of the available resources (posters, websites, toolkits).

Examination of baseline level of organizational capacity (second objective) was conducted prior to the official unveiling of the 'new' ParticipACTION. At that point, the full details of the collaborative initiatives and process between ParticipACTION and its partners and stakeholders had not been identified. Despite this lack of specific detail, organizations in general reported that they have good capacity to integrate new physical activity campaigns -- such as those led by ParticipACTION -- into their mandates. Overall capacity means ranged from approximately 3.7 to 4.1 on 5-point scales. The high capacity may be due to a ceiling effect, which may be due to response bias. Since 85% of the responding organizations have been operating for over 10 years, there may be little need or room to expand capacity. Although the analysis did not specifically examine organizational longevity, it could be argued that this may positively influence capacity. It is also possible the high capacity is due to innovation-values fit, where the values of the organization surveyed and the vision of ParticipACTION are compatible [25]. Results also show little variation in capacity to adopt, implement or externally promote a new physical activity campaign across organizational size, sector or mandate.

Regarding the third objective, exploring differences in organizational capacity, no significant findings emerged to suggest a relationship between size or type of organization and increased capacity of integrating initiatives like those led by ParticipACTION. There were, however, three interesting and possibly relevant observations. We found that within an organization's capacity to adopt a new physical activity initiative, there was a significant difference between the government and not-for-profit sectors. Government, which includes educational institutions, appears to have a higher capacity to adopt a new initiative than not-for-profit organizations. Two possible reasons for this are: (1) greater financial resources; and, (2) greater human resources in the government sector as compared to the not-for-profit organizations to deal with externally generated initiatives [21]. The issues of budgets, funding, and human resources were not included in this survey but should be investigated in future studies of organizational capacity for physical activity promotion.

We also found two significant differences in organizations' capacity to adopt and implement based on mandate. The data show that educational organizations (such as school boards) have greater capacity to implement an initiative than do public health/health care organizations or sport and sport/recreation organizations. There are a number of possible reasons why educational organizations have greater capacity than either health or sport organizations to adopt, implement and promote physical activity. It may be that educational organizations have: (1) better organizational structure and physical facilities; (2) more trained personnel within the schools to deliver programs; (3) higher capacity to interact with groups and providers outside the school environment to develop and deliver programs; and, (4) greater capacity to reach most children and adolescents due to the nature of the educational system [26]. It is also possible educational organizations have greater capacity to adopt a new initiative (consistent with DIT) because: (1) they see a relative advantage of the innovation; (2) the innovation is compatible with their needs; (3) it is not overly complex to understand and implement the innovation; and, (4) an innovation can be assessed and observed on an ongoing basis [27]. It may also be that educational organizations see ParticipACTION's broad goal of promoting both physical activity and health meshing with their own goals.

Organizations' decision to work with ParticipACTION may depend on the "fit" between ParticipACTION's campaign and their own organizational vision and values [25,28]. Both health care/promotion and sport/recreation organizations may see the general merit of ParticipACTION's efforts. They may not, however, see the benefit of that campaign to their specific organization. For example, sporting groups may acknowledge ParticipACTION's message, but may not see an advantage to their organization in adopting something that already exists within their own organization, namely physical activity programs. They may view this as a duplication of effort, or an intrusion into their existing work. Health care groups, on the other hand, may perceive the advantage of promoting the health benefits of physical activity, but may only be in a position to offer advice and information about it, not programs. They may feel they already offer enough information.

There may also be a lack of infrastructure and resources to collaborate with and to meet the demands of an organization like ParticipACTION. Implementing and sustaining a health promotion campaign within the sport and recreation sector, or implementing and sustaining a physical activity campaign within the health care sector may require additional resources. These resources may be scarce. It may also require new approaches and the creation of new organizational values to support the efforts of the 'new' ParticipACTION [28].

Evidence of some variation on the basis of organizational sector or mandate points to an area requiring future inquiry. The science behind physical activity promotion is growing, but the practice at the population level is lagging, with the infrastructure for physical activity promotion for public health practice being both underdeveloped and untested [29]. ParticipACTION's primary mandate is to promote a more active Canada, across all regions and all populations. It will do this through the channels it develops with its partners, and through the organizational capacity of those partners. What kinds of messages will ParticipACTION create given the diversity of these organizations? Will messages and information be designed to reflect the size, sector and mandate of the organizations in its network or will it try to create generic/universal messages? How will ParticipACTION disseminate the information? How will it monitor the efficacy of those communications? For successful initiation and then sustainability of a campaign, there needs to be a strategy for both dissemination and diffusion of information [15]. It appears the time is right for developing infrastructures within the public health field that will promote physical activity and its impact on the well-being of individuals and societies [29], in the same way that public health infrastructures have been developed for other major health concerns [29].

We acknowledge a number of limitations to our study. First, although the response rate of 29.7% is reflective of on-line survey results [30-32], the study may have missed some alliances, networks, organizations, institutions and businesses. Due to the diversity of both the Canadian population and the types of organizations that deliver physical activity related information (or are in some way peripherally involved with its promotion), it is difficult to generate a list which accurately represents the sectors (government, not-for-profit, private) and mandates (education, health, sport, and recreation) across Canada. Although there is no existing database from which to draw names of organizations, we made every effort to be as inclusive and representative as possible. It is also possible that some invited organizations did not feel they met the criteria for the study and therefore chose not to participate.

We received a good response rate from organizations with certain primary mandates while response rates for others were lower. Just under half (48%) of respondents identified education as their primary mandate (we contacted 281 school boards across the country, or 31% of invitees) providing us with an over-representation by education. Just over one-third (34.1%) of respondents identified sport and/or recreation as their primary mandate (217 sport organizations, or 24% of invitees). Only 10.3% of respondents identified public health/health care as their primary mandate (approximately 360 organizations were contacted or 41% of invitees), a low response rate that may be due to the fact that some of these organizations see themselves as having multiple mandates. For example, an organization such as YMCA offers education, health, sport and social support programs [33]. It is also possible the definition of health care and promotion was too broad while respondents perceived the term too narrowly. Future studies should take into account multiple mandates. We may have improved our response rate by contacting invitees prior to sending the survey invitation [34].

Second, we had a very low response rate of 1.6% (3 responses) from the private sector (39 invitations, or 4% of invitees) which included fitness facilities, manufacturers and distributors. One reason for the low response may be accessibility; identifying and contacting specific individuals within companies via email was difficult. Internet communication often directed us to customer relations. It is, however, important to secure feedback from the private sector. This sector, with its myriad stakeholders, may be in a position to help promote the goals of ParticipACTION while concurrently expanding their business opportunities [35]. ParticipACTION may successfully align itself with private sector companies, such as the *Canada on the Move *initiative did with *Kellogg's *in pedometer distribution [36]. Companies may pursue co-branding with ParticipACTION or other related organizations. In future, accessing the private sector for a survey could be approached differently, using a combination of postal, *voice *and internet modes of contact [32]. With the increase in work-based physical activity programs and private companies which provide these services, we may be able to expand our private sector sample to include corporate health/wellness organizations and their clients.

Third, the number of organizations responding to the survey generally reflected Canada's population by province, with the exception of Quebec and British Columbia where apparently fewer organizations contacted actually completed the survey. Since to our knowledge there is no census of organizations promoting physical activity in Canada, it is difficult to say whether or not these apparently differential patterns of response are actual deviations from representativeness. Caution is thus required in generalizing the findings reported here nationally.

## Conclusion

The ability of organizations to effectively implement and disseminate broad-based national campaigns and strategies to mobilize physical activity initiatives has been slow, small-scale, short-term and, in many cases, fragmented [37]. With the exception of countries like Canada and Finland, there is little evidence such campaigns have worked [37]. Even in Canada, which has demonstrated success with wide-ranging physical activity programs and increases in participation over the years [38,39], rates of inactivity and obesity are rising among segments of the general population [40], particularly children [41,42]. These patterns may be caused by a lack of capacity at the ministerial level (health, sport, education) and/or relevant non-governmental organizations' ability to respond to the needs of physical activity initiatives [37]. At the international level, a number of barriers to promoting physical activity initiatives have been identified including a lack of government support; low profile and poor understanding of physical activity and its impact; lack of infrastructure; lack of leadership; inexperience in partnerships; competing demands; lack of resources; and, limited program guidelines and training [37].

Due to the strong branding of ParticipACTION (old and new) combined with the capacities of organizations and advances in communication technology, there is great potential for strong partnerships to promote a more physically active Canada. This study adds to the growing body of knowledge on organizational capacity and physical activity initiatives by showing that Canadian organizations involved in physical activity and health promotion have a good capacity to work with a new communications leader in this area. The majority of organizations responding to this study, regardless of size, sector, or mandate, report they have the capacity to work on externally created campaigns that may be spearheaded by the 'new' ParticipACTION.

We now have a national baseline assessment of organizational capacity that allows for an ongoing, long-term evaluation of the impact of ParticipACTION. This assessment took place before ParticipACTION began disseminating information and before its partners implemented any changes to adopt and integrate the campaign into their mandates. This baseline now provides us with the unique opportunity for future studies to assess the impact of 'new' ParticipACTION. For example, we will be able to determine whether or not organizations chose to work with ParticipACTION, why they chose to work with ParticipACTION (or why they chose not to), and what organizational changes they needed to make (if any) to allow them to do so. We can also determine whether a particular sector (government, not-for-profit), mandate (education, sport/recreation, health care/health promotion) or size (small, medium, large) is better suited to adopting a new idea. Future work may also look at how communications are tailored and then disseminated to meet the requirements and resources of organizations based on sector and mandate to optimize adoption, implementation and external promotion of physical activity initiatives such as ParticipACTION.

## Appendix 1

Scales to measure organizational capacity to adopt, implement and externally promote physical activity.

Organizational capacity to adopt a new PA initiative* (α = .89)

Which of these factors would play a part in adopting new Physical Activity initiatives within your organization?

(1) A strong vision of organizational goals and strategies.

(2) Organizational leadership.

(3) "Internal champion".

(4) Managerial practices.

(5) Organizational structure.

(6) Knowledge sharing procedures and infrastructure.

(7) Coordination of programs within the organization.

Organizational capacity to implement a new PA initiative* (α = .90)

To what extent are the following factors true about your organization?

(1) The organizational vision is updated in response to changes in the environment.

(2) Employees take into account the organization's vision as they execute their work.

(3) Our organization's leader(s) demonstrates a responsive and accessible style.

(4) Our organization devotes adequate time to long-range planning.

(5) Our organization has a good idea of where it wants to be in 5 years.

(6) Our organization fosters a learning culture and climate.

(7) Processes are in place for group decision-making.

(8) Processes are in place for evaluating past decisions.

(9) We are quick to meet challenges in our environment.

(10) Honesty and trustworthiness characterize the relationships within our organization.

(11) We provide training opportunities for employees within our organization.

Organizational capacity domain to externally promote a new PA initiative* (α = .90)

To what extent does each of these factors play a part in externally promoting a new PA initiative by your organization?

(1) Organizational leadership.

(2) "Internal champion".

(3) Managerial practices.

(4) Board initiative.

(5) Organizational structure.

(6) Infrastructure (e.g., budget).

(7) Knowledge sharing procedures.

(8) Partnerships with other agencies or groups.

(9) Media connections.

* 5-point Likert-type scale: (1) = 'not at all'; (5) = 'very'

## Competing interests

The authors have been involved on various committees and advisory groups for both the previous and 'new' ParticipACTION including the Board of Directors (MT). The Canadian Fitness and Lifestyle Research Institute (CFLRI) was previously contracted by the 'new' ParticipACTION to perform research and surveillance - CLC is President of CFLRI but received no personal compensation for the contracted work.

## Authors' contributions

RCP and GF conceived the study. RCP, IT, GF, SC and CLC were involved in the survey design. IT coordinated survey delivery and data collection. MAP and RCP analyzed and interpreted the data. RCP and IT wrote all drafts of the manuscript. All authors (RCP, IT, GF, MAP, SC, KC, CLC, JCS, MT, AB, LB and LG) contributed to the concept and design of the study, critically evaluated the article for content and approved the final version.

## Acknowledgements

1. This project was made possible by a grant from Canadian Institutes of Health Research.

2. We acknowledge the work of Nicole Glenn of the Faculty of Physical Education and Recreation at the University of Alberta in reviewing this paper.

3. RCP holds a Canadian Institutes of Health Research Applied Public Health Chair in Physical Activity and Public Health

4. LB holds a Tier 1 Canada Research Chair in Physical Activity in Health Promotion and Disease Prevention.

5. LG holds a Canadian Institute for Health Research/Centre de Recherche en Prévention de l'Obésité Applied Public Health Chair in Neighborhoods, Lifestyle, and Healthy Body Weight.
